# Opposites attract? Mixed‐sex parents' and siblings' sport participation differentiates youth athletes' perceived parenting climates

**DOI:** 10.1002/ejsc.12112

**Published:** 2024-04-20

**Authors:** Tsz Lun (Alan) Chu, Brett J. Garst

**Affiliations:** ^1^ Department of Kinesiology University of North Carolina at Greensboro Greensboro North Carolina USA; ^2^ Department of Psychology University of Wisconsin—Green Bay Green Bay Wisconsin USA

**Keywords:** family systems, gender socialization, modeling, motivational climates

## Abstract

Guided by family systems and achievement goal theories, this study examined how the sex of athletes and their main sport parents, as well as sport participation patterns (same sport, different sports, and no sports) of parent–athlete and sibling sex compositions (same‐sex and mixed‐sex), differentiated athlete perceptions of parenting climates—task‐involving (emphasizing individual improvements, effort, and mastery) and ego‐involving (emphasizing winning and performance comparison). Participants were 353 U.S. high school athletes (*M*_age_ = 15.52 and *SD* = 1.18; 55% male) who completed a survey on perceived parenting climates, family compositions, and sport backgrounds of their parents and siblings. We conducted six moderated regression analyses, two of which used (1) athlete sex and main sport parents' sex, (2) sport participation patterns of parent–athlete sex compositions, or (3) sport participation patterns of sibling sex compositions as independent variables. Four of the analyses were statistically significant with small effect sizes, showing that (1) boys perceived greater ego‐involving climates than girls; (2) athletes whose same‐sex parents played sports (same or different sports) compared to no sports‐perceived greater task‐involving climates: (3) athletes whose mixed‐sex parents played (same or different sports) compared to no sports‐perceived greater task‐involving climates and less ego‐involving climates; and (4) athletes whose mixed‐sex siblings played different sports than they did, compared no sports, and perceived greater task‐involving climates. None of the interactions were significant. Findings provide theoretical and practical implications by incorporating motivational climates, addressing the potential relationships of parents' and mixed‐sex siblings' sport participation to adaptive parenting climates.

## Highlights

- Family system, including parents and siblings, and their sex compositions are important factors to consider when examining athlete perceptions of parenting.
- Athletes perceived greater task‐involving when their same‐sex or mixed‐sex parents played sports rather than no sports and less ego‐involving climates when their mixed‐sex parents played sports rather than no sports.
- Athletes perceived greater task‐involving climates when their mixed‐sex siblings played different sports than they did rather than no sports.

## 1 INTRODUCTION

Youths are constantly exposed to their parents' success standards and interpret them based on parental behavior and expectations in various social contexts, including sport (Fredricks & Eccles, 2005). Due to the significant amounts of time spent with their families, youth athletes' perceived motivational climates created by their parents influence their sport participation processes and outcomes, such as goal orientation (Gomes et al., 2019; White, 1996). Achievement goal theory (Ames, 1992) categorizes these perceptions of competence and success standards, created by social agents such as coaches and parents (White, 1996), into two types of motivational climates—*task‐involving*, emphasizing individual improvements, effort, and mastery, and *ego‐involving*, emphasizing winning and performance comparison. Although sport motivation researchers have studied parenting climates extensively (see Chu & Zhang, 2019; Harwood et al., 2015), they only recently took into account sibling influence (see Dorsch et al., 2021), a critical part of the family system.

The family system, including intergenerational (e.g., parents) and intragenerational relationships (e.g., siblings), is crucial for the development of identities and interests throughout childhood and adolescence (McHale et al., 2003, 2012). Youths tend to identify more closely with their same‐sex than mixed‐sex parents; girls and boys more frequently approach the mother and the father, respectively, to fill their needs and develop interests, including sport (Raley & Blanchi, 2006). Studies have indicated mothers as the primary facilitator for girls' sport involvements and fathers as the predominant socializing agent for boys'. Contemporary research, however, suggests that both mothers and fathers actively engage in youth sport communities to encourage their children's participation (see Dorsch et al., 2021). Such engagements can be analyzed and explained through family systems theory (FST). FST describes the family unit as a system made up of smaller subsystems that, if changed, will lead to compensatory changes in other subsystems and the overall family system (Bowen, 1966). Another key component of FST is the degree to which individuals within the family differentiate themselves from the family, ranging from differentiation to ego fusion (Bowen, 1966). Ego fusion—little differentiation of self—signifies modeling behaviors based on perceived roles within the family system (Pahl & Spencer, 2010). Such behaviors and roles are often influenced by family sex composition.

Grounded in FST and empirical evidence, the gendered family process (GFP; Endendijk et al., 2018) model explains biological, social, and behavioral impacts on gender development. A key component of the GFP model is the relationships among family sex composition, gender cognitions within the family, and gender‐stereotyped behaviors by the child. The family sex composition affects the cognitions regarding gender and gender‐stereotyped behaviors (masculine vs. feminine) within the family, which in turn influences the amount of gender‐stereotyped behaviors exhibited by the child and their siblings (Endendijk et al., 2018). Family sex composition includes both parent and sibling sex compositions (same‐sex and mixed‐sex siblings) that play a role in familial gendered cognitions and behaviors (Endendijk et al., 2018). The literature suggests that the presence of same‐sex siblings reinforces gender‐typical cognitions and behaviors (Endendijk et al., 2018). In contrast, the presence of mixed‐sex siblings in the family has a gender‐neutralizing effect on parental behaviors and stereotypes, with more egalitarianism through differential familial roles and deidentification (Endendijk et al., 2013). The family sex composition has a prominent effect later in adolescence due to the identity formation phase (Schachter & Galliher, 2018). Taken together, family sex composition can influence gendered cognitions, which likely affect youth perception of parenting climates.

Parents' experiences, knowledge, and goals in sport influence the parenting climates that they create and that their children perceive through socialization and modeling (Fredricks & Eccles, 2005; Gomes et al., 2019). For instance, parents use their sport expertize to gain compliance from their child athletes, who perceive the climate as positive if parents show support and task orientation (Turman, 2007; White, 1996). In addition to parents' sport participation, siblings' sport participation might also influence parenting climates based on FST (Minuchin, 1974), accounting for factors within and across individuals in a family (Dorsch et al., 2022). Sibling influence is well‐documented in the literature on family studies (McHale et al., 2003, 2012) and achievement contexts such as education (Whiteman et al., 2007). However, it has been understudied in sport psychology (Blazo & Smith, 2018; Dorsch et al., 2022), even though siblings appear to be a significant socializing agent influencing a child's sport participation and interests (Osai et al., 2020; Osai & Whiteman, 2017). Blazo and Smith's (2018) systematic review of sibling influence in sport and physical activity contexts indicated the importance of applying social learning and motivation theories to examine sibling modeling and rivalry. They further noted that although the findings on sex composition were inconclusive, same‐sex siblings were more likely to express warmth and be influenced to participate in sport.

More recently, Osai et al. (2020) implemented a family systems approach to examine how siblings' sex composition, age difference, modeling and differentiation (i.e., pursuing similar vs. different activities), and perceived mothers' and fathers' behaviors predicted the likelihood of the siblings playing the same primary sport. Mixed‐sex composition and sibling differentiation predicted a lower likelihood of playing the same primary sport, whereas sibling modeling and parental behaviors were not significant predictors. To design and implement interventions, however, it might be more important to study the reverse—how siblings' sport participation patterns predict athlete perceptions of parenting. For instance, collegiate athletes in a qualitative study expressed feeling pressure from their family members and community, and in turn a sense of competition and jealousy (i.e., ego‐involving climates), when their siblings were also collegiate athletes (Blazo et al., 2014). Yet, sibling sex and participation in the same or different sports were not reported, and no published studies considered the role of siblings who did not play sports. Similarly, although the significant modeling roles of mothers' and fathers' sport participation have been examined in various studies (see Dorsch et al., 2021), none to our knowledge have considered how their sport participation patterns (i.e., playing the same sport as the child, different sports, or no sports) might play a role in perceived parenting climates. Understanding these relationships in same‐sex and mixed‐sex parent–child or sibling composition could help practitioners tailor their motivational interventions to address specific family dynamics and sport backgrounds instead of using a one‐size‐fits‐all approach with youth athletes and their parents. Examining athletes' (vs. parents') perceptions by sex composition would be particularly informative because parents tend to view their behavior as more supportive than do their children (Kanters et al., 2008).

Due to these gaps in research and practice, this study aimed to provide insights into how sport participation of same‐sex and mixed‐sex composition within the family might be related to perceptions of parenting climates by examining the role of socializing agents' sex and sport participation patterns. Two research questions were proposed: (1) How may youth athletes' and their main sport parents' (i.e., the parent most involved in the athlete's sport participation) sex differentiate athlete perceptions of parenting climates? (2) How may parent–athlete and sibling sex compositions’ (i.e., same‐sex and mixed‐sex) sport participation patterns (same sport, different sports, and no sports) differentiate youth athletes' perceptions of parenting climates?

## 2 METHOD

### 2.1 Participants and procedure

The initial sample consisted of 383 high school athletes, whose primary sport included baseball, basketball, cross country, American football, soccer, tennis, track and field, volleyball, and softball, in the southwestern and midwestern U.S. Twenty‐seven athletes reported “not applicable” regarding their father's or mother's sport participation, and three outliers were detected (see descriptions below). Upon removal of the data, the final participants were 353 athletes (*M*_age_ = 15.52 and *SD* = 1.18; 55% male and 59.5% White). This sample was larger than the minimum sample size (*N* = 153) calculated based on a priori power analysis via G*Power—*F* tests for multiple regression using Bonferroni‐adjusted *α* = 0.0167, power = 0.80, and medium effect size *ƒ* = 0.15. Following the Institutional Review Board approval and school permission, we obtained informed parental consent and child assent before administering the paper‐and‐pencil survey to the participants in a classroom during each team's practice time.

### 2.2 Measures

The survey items included demographic questions, sport backgrounds (e.g., primary sport), family structure (e.g., number of brothers and sisters), family sex composition, and questions on their family members' sport participation patterns (“Do/Did your parents/siblings play sports competitively?”). Responses for each role (father, mother, brother, and sister) include (1) *yes, the same sport as mine*, (2) *yes, a different sport than mine*, (3) *no*, and (4) *does not apply*. In addition, participants completed the 18‐item Parent‐Initiated Motivational Climate Questionnaire (PIMCQ‐2; White, 1996) with respect to the perceived climates from their reported main sport parent (father, mother, and other). Using a Likert scale ranging from 1 (*strongly disagree*) to 5 (*strongly agree*), participants responded to the stem “I feel that my parent…” and the items for three subscales: Learning/Enjoyment (e.g., “encourages me to enjoy learning new skills”), Worry‐Conducive (e.g., “makes me worried about failing”), and Success Without Effort (e.g., “thinks I should achieve a lot without much effort”).

### 2.3 Data analysis

First, we screened the data for invalid and missing values, outliers (|z| > 3), and normality (Tabachnick & Fidell, 2013). Only three parenting climate items (0.04% of all values) were missing in three different participants; thus, we employed person mean substitution (Hawthorne & Elliott, 2005). The data were normally distributed with skewness and kurtosis between −2 and 2. To compute the dependent variables, we averaged the Learning/Enjoyment subscale items to form task‐involving climate scores and averaged the Worry‐Conductive subscale and Success Without Effort subscale items to form ego‐involving climate scores, both demonstrating good internal reliability (*ω* = 0.83 and 0.75, respectively). To form the independent variables, we first categorized parents' and siblings' sport participation patterns into three levels (same, different, or no sports) for each of the four sex compositions: (1) same‐sex parent–athlete (i.e., mother–daughter or father–son), (2) mixed‐sex parent–athlete (i.e., mother–son or father–daughter), (3) same‐sex sibling (i.e., brother–brother or sister–sister), and (4) mixed‐sex sibling (i.e., sister–brother). We then dummy coded these variables using “no sports” as the reference groups.

Regarding the main analyses, we conducted six moderated regression analyses using Model 1 of the PROCESS macro Version 4.3.1 (Hayes, 2022), with task‐involving or ego‐involving climates as the dependent variable in each analysis. To answer the first research question, two of the analyses used athlete sex, main sport parents' sex, and the interaction between the two as the independent variables. To answer the second research question, two analyses used the dummy‐coded same‐sex parent–athlete sport participation patterns, mixed‐sex parent–athlete sport participation patterns, and the interaction between the two as the independent variables. Finally, two analyses used dummy‐coded same‐sex sibling sport participation patterns, mixed‐sex sibling sport participation patterns, and the interaction between the two as the independent variables. Athlete sex was used as a covariate due to its theoretical and statistical significance in differentiating parenting climates (Dorsch et al., 2021). To reduce Type I errors, Bonferroni‐adjusted *α* = 0.0167 (0.05/3) was used to determine the statistical significance of the regression models. Within statistically significant regression models, a 95% bias‐corrected confidence interval (CI) that does not contain zero based on 5000 bootstrap samples indicates statistically significant predictors (Hayes, 2022). Effect sizes were determined using *R* ^2^ = 0.02, 0.13, and 0.26 for small, medium, and large effects, respectively (Cohen, 1988).

## 3 RESULTS

The results of the six regression analyses are displayed in Table 1. None of the interactions were significant in predicting parenting climates as indicated by *∆R* ^2^ in Table 1; therefore, we did not include the description of relevant statistics in the results section. For the regression analyses with athlete sex and main sport parents' sex as the independent variables, the overall model for predicting task‐involving climates was not significant, *F*(3, 318) = 1.631, *p* = 0.182. On the other hand, the overall model for predicting ego‐involving climates was significant, *F*(3, 318) = 3.784, *p* = 0.011, accounting for 3.5% of the variance. Athlete sex was the only significant predictor, indicating perceptions of more ego‐involving climates in boys than girls.

**TABLE 1:** Moderated regression analyses of sex compositions and sport participation patterns predicting parenting climates.

Regression models	*F*	*R* ^2^	*∆R* ^2^	*B*	*t*	LLCI	ULCI
Model 1 DV: Task‐involving climates	1.631	0.015	0.003				
Athlete sex				−0.256	−1.186	−0.682	0.169
Main parent sex				−0.204	−2.014	−0.404	−0.005
Model 2 DV: Ego‐involving climates	3.784*	0.035	0.006				
Athlete sex				**0.553**	2.284	0.767	1.029
Main parent sex				0.214	1.888	−0.009	0.438
Model 3 DV: Task‐involving climates	4.318*	0.102	0.014				
Same‐sex parent–athlete, same sport				**0.355**	3.073	0.128	0.583
Same‐sex parent–athlete, different sports				**0.334**	2.815	0.100	0.563
Mixed‐sex parent–athlete, same sport				**0.615**	3.132	0.229	1.002
Mixed‐sex parent–athlete, different sports				**0.385**	3.157	0.145	0.625
Model 4 DV: Ego‐involving climates	3.093*	0.075	0.020				
Same‐sex parent–athlete, same sport				0.086	0.638	−0.180	0.352
Same‐sex parent–athlete, different sports				−0.256	−1.423	−0.466	0.075
Mixed‐sex parent–athlete, same sport				**−0.537**	−2.338	−0.989	−0.085
Mixed‐sex parent–athlete, different sports				**−0.331**	−2.320	−0.611	−0.050
Model 5 DV: Task‐involving climates	2.378*	0.100	0.019				
Same‐sex siblings, same sport				−0.032	−0.199	−0.351	0.287
Same‐sex siblings, different sports				0.383	1.931	−0.008	0.774
Mixed‐sex siblings, same sport				0.230	0.987	−0.230	0.690
Mixed‐sex siblings, different sports				**0.402**	2.198	0.041	0.763
Model 6 DV: Ego‐involving climates	2.321	0.098	0.007				
Same‐sex siblings, same sport				−0.182	−1.024	−0.531	0.168
Same‐sex siblings, different sports				−0.262	−1.205	−0.690	0.167
Mixed‐sex siblings, same sport				−0.487	−1.907	−0.991	0.017
Mixed‐sex siblings, different sports				−0.081	−0.402	−0.476	0.315

*Note*: *∆R* ^2^ = variance explained by the (nonsignificant) interactions of independent variables. B = unstandardized regression coefficient. LLCI = 95% lower limit confidence interval; ULCI = 95% upper limit confidence interval. **Bolded** coefficients indicate significant predictors. *p < 0.0167.

For the regression analyses with the sport participation patterns of same‐sex and mixed‐sex parent–athlete compositions as the independent variables, the overall model for predicting task‐involving climates was significant, *F*(9, 343) = 4.318, *p* < 0.001, accounting for 10.2% of the variance. The overall model for predicting ego‐involving climates was also significant, *F*(9, 343) = 3.093, *p* = 0.001, accounting for 7.5% of the variance. Taken together, same‐sex parent–athlete compositions participating in the same sport and different sports were both significant predictors, indicating perceptions of more task‐involving climates in athletes with these compositions and patterns compared to those whose same‐sex parents did not participate in any sport. Furthermore, mixed‐sex parent–athlete compositions participating in the same sport and different sports were both significant predictors, indicating perceptions of more task‐involving and less ego‐involving climates in athletes with these compositions and patterns compared to those whose mixed‐sex parents did not participate in any sport. Table 2 shows the descriptive statistics of these parenting climate mean comparisons across parent–athlete compositions.

**TABLE 2:** Descriptive statistics of perceived parenting climates across parent–athlete and sibling compositions.

		Same sport		Different sports		No sports	
		M	SD	M	SD	M	SD
Same‐sex parent–athlete	Task‐involving climates	4.12_a_	0.52	4.05_b_	0.52	3.80_ab_	0.69
	Ego‐involving climates	2.51	0.73	2.50	0.65	2.51	0.69
Mixed‐sex parent–athlete	Task‐involving climates	4.22_a_	0.31	4.09_b_	0.57	3.82_ab_	0.64
	Ego‐involving climates	2.27_a_	0.71	2.42_b_	0.68	2.66_ab_	0.67
Same‐sex siblings	Task‐involving climates	3.95	0.63	4.14	0.52	3.83	0.66
	Ego‐involving climates	2.58	0.62	2.47	0.67	2.66	0.75
Mixed‐sex siblings	Task‐involving climates	4.06	0.54	4.08_b_	0.59	3.78_b_	0.64
	Ego‐involving climates	2.26	0.74	2.59	0.65	2.69	0.64

*Note*: Range = 1–5 for tasking‐involving and ego‐involving climates. Means with the same lettered subscripts significantly differed based on the results of regression analyses. Respective proportions of socializing agents playing the same sport as the athlete, different sports, and no sports: (1) same‐sex parents (34.0%, 32.6%, and 33.4%); (2) mixed‐sex parents (9.9%, 47.6%, and 42.5%); (3) same‐sex siblings (38.9%, 30.5%, and 30.5%); (4) mixed‐sex siblings (16.7%, 47.3%, and 36.0%).

For the regression analyses with the sport participation patterns of same‐sex and mixed‐sex sibling compositions as the independent variables, the overall model for predicting task‐involving climates was significant, *F*(9, 193) = 2.377, *p* = 0.014, accounting for 10.0% of the variance. However, the overall model for predicting ego‐involving climates was not significant, *F*(9, 193) = 2.321, *p* = 0.017. Mixed‐sex sibling compositions participating in different sports was the only significant predictor, indicating perceptions of more task‐involving climates in athletes with these compositions and patterns compared to those whose mixed‐sex siblings did not participate in any sport (see Table 2).

## 4 DISCUSSION

This study expanded the sport motivation literature by using a family systems approach to examine the role of parents' and siblings' sex and sport participation patterns in youth athletes' perceptions of parenting climates. Similar to previous findings indicating greater compliance‐gaining techniques from boys' than girls' parents due to sex‐role socialization (Turman, 2007), boys in our study perceived greater ego‐involving climates than girls with a small effect size. On the other hand, perceived parenting climates from mothers and fathers as the main sport parent did not differ, supporting recent research that found relatively similar parental behaviors of both mothers and fathers involved in their children's sport participation (Dorsch et al., 2021). Our findings coincide in part with the notion from FST and the GFP model that the presence of mixed‐sex siblings in a family unit changes the family dynamics by promoting more egalitarian cognitions and behaviors. Greater task‐involving parenting climates were reported by athletes whose mixed‐sex siblings played different sports than they did compared to no sports. When mixed‐sex siblings play sports, athletes may experience a gender‐neutralizing effect that reduces parental gender stereotypes for their children (Endendijk et al., 2018). More specifically, participating in different sports between siblings likely contributes to greater emphases on individual improvements and mastery instead of performance comparisons between the siblings.

Concerning parent–athlete sex compositions' roles in perceived parenting climates, the significant roles of same‐sex parents' sport participation patterns align with social learning theory and relevant research on same‐sex parents' significant modeling roles (Perry & Bussey, 1979; Raley & Blanchi, 2006). At the same time, the significant roles of mixed‐sex parents' sport participation patterns support both mothers' and fathers' active engagement and encouragement of their children's sport participation in recent years (Dorsch et al., 2021). These results suggest that youth athletes, regardless of their sex, generally perceive their parents to focus more on learning and enjoyment than winning and outperforming others when the parents (especially the mixed‐sex) play(ed) sport. A potential explanation is that parents who play(ed) sports may be more involved in athletes' sport activities to provide modeling of performance standards for individual improvements and mastery (Perry & Bussey, 1979). Further, consistent with FST that emphasizes the complementarity of family members, more involvement and direction from the mixed‐sex parent who has a sport background might help balance out parental involvement of the same‐sex parent, thus reducing perceptions of same‐sex parents' behaviors that could be ego‐involving. This explanation is in line with Lienhart et al.’s (2019) recent finding that mothers' active involvement and pressure and fathers' directive behaviors predicted adaptive motivation only in mixed‐sex youth athletes.

In terms of sibling sex compositions' roles in perceived parenting climates, although no significant differences were found across same‐sex siblings' sport participation patterns, the athletes perceived greater task‐involving climates when their mixed‐sex siblings played different sports than they did compared to no sports. Sibling modeling and deidentification could potentially explain these differential levels of perceived parenting climates, especially since mixed‐sex siblings tend to express less modeling behaviors and warmth than do same‐sex siblings (Blazo & Smith, 2018; Whiteman et al., 2007). Specifically, when mixed‐sex siblings participate in sport, it might (a) enhance sibling modeling, which is associated with less sibling conflict and more parental warmth, and (b) reduce sibling deidentification, which is related to more sibling and parental conflict and less parental warmth (Osai et al., 2020). In addition to perceiving more parental warmth and less conflict, having a mixed‐sex sibling who plays a different sport might draw more balanced involvement from parents with less sibling comparisons; thus, the athlete may perceive a stronger parental emphasis on task mastery and enjoyment over achievements in sport (Lienhart et al., 2019). Supporting the complementarity of siblings based on FST and the GFP model, the mere presence of a mixed‐sex sibling within the home has been shown to reduce gender‐stereotyped expectations and cognitions in parents, thus aiding in the development of a task‐involving climate (Endendijk et al., 2018).

The novel findings from our study provide preliminary insights into assessing the motivational influence of parent–athlete and sibling sex compositions. The main implication is for practitioners (e.g., mental performance consultants, psychologists) to consider the holistic family system when working with athletes or parents on improving motivational climates and associated outcomes, such as goal orientation and anxiety (Dorsch et al., 2021). When conducting need assessments and planning interventions, especially for athletes who seem to struggle with motivation or fear of failure, practitioners should implement formal written or verbal assessments of athlete families' structure, sex composition climates, and sport backgrounds beyond the typical assessments of coaching and teammate influence (Chu & Zhang, 2019; Dorsch et al., 2022). Although a multitude of factors could influence parenting climates, our findings prompt practitioners to pay particular attention to athletes with mixed‐sex parents or siblings who do not play sports. To intervene with perceived maladaptive patterns of parenting climates (i.e., high ego‐involving and low task‐involving), practitioners may teach athletes adaptive cognitive appraisal to interpret parental expectations and pressure to win as challenges rather than threats (Gomes et al., 2019). Practitioners can also encourage athletes and parents to discuss their perceptions of parenting climates, as well as any inconsistencies, with mutually agreed solutions to improve them (Kanters et al., 2008). In a similar vein, the potential influence of siblings' sport (non)participation on perceived parenting climates should be discussed. Furthermore, practitioners ought to educate parents about how sex composition and modeling versus deidentification among siblings might be related to athletes' sport experience and perceived motivational climates and, in turn, how parents should adjust their parenting practices (Blazo & Smith, 2018; McHale et al., 2003).

Despite the novel findings and implications, our study has limitations. Intended to be a preliminary investigation with adequate statistical power, this study did not assess and analyze sibling positions (e.g., oldest, youngest), age differences, and warmth and conflict that have been shown influential in the sibling modeling and deidentification processes (Osai et al., 2020; Whiteman et al., 2007). Future research incorporating these factors could provide a more complete picture of how same‐sex and mixed‐sex siblings' sport participation patterns are related to sport parenting. Moreover, this study was conducted in a U.S. high school sport context, which could not be generalized to other age groups or countries, especially in cultures where family and sport values are very different. More importantly, due to the logistics of the study and the preliminary nature of the analyses, we could only assess sex and sex compositions but not gender identities and other types of family compositions (e.g., two fathers/mothers and stepparents). Further examining various types of family compositions and systemic factors that influence family socialization, sport parenting, and sibling relationships would help researchers understand the nuances within family systems and practitioners work more effectively with diverse athletes and families (Dorsch et al., 2022).

In conclusion, our study suggests that youth athletes generally perceive more task‐involving parenting climates when their same‐sex or mixed‐sex parents play(ed) sports and when their mixed‐sex siblings play different sports than they did, compared to those whose parents and siblings do/did not play sports, respectively. These results fill the literature and add to our understanding of parenting climates using a family systems approach. Researchers and practitioners alike should continue to consider various family socialization factors that influence athletes' motivational processes across developmental stages.

## CONFLICT OF INTEREST STATEMENT

No conflict of interest to disclose.
